# Patterns of home care assessment and service provision before and during the COVID-19 pandemic in Ontario, Canada

**DOI:** 10.1371/journal.pone.0266160

**Published:** 2022-03-30

**Authors:** Chi-Ling Joanna Sinn, Heebah Sultan, Luke Andrew Turcotte, Caitlin McArthur, John P. Hirdes

**Affiliations:** 1 School of Public Health Sciences, University of Waterloo, Waterloo, Ontario, Canada; 2 Institute of Health Policy, Management and Evaluation, University of Toronto, Toronto, Ontario, Canada; 3 Ontario Health, Toronto, Ontario, Canada; 4 School of Physiotherapy, Dalhousie University, Halifax, Nova Scotia, Canada; Ehime University Graduate School of Medicine, JAPAN

## Abstract

**Objective:**

The objective was to compare home care episode, standardised assessment, and service patterns in Ontario’s publicly funded home care system during the first wave of the COVID-19 pandemic (i.e., March to September 2020) using the previous year as reference.

**Study design and setting:**

We plotted monthly time series data from March 2019 to September 2020 for home care recipients in Ontario, Canada. Home care episodes were linked to interRAI Home Care assessments, interRAI Contact Assessments, and home care services. Health status measures from the patient’s most recent interRAI assessment were used to stratify the receipt of personal support, nursing, and occupational or physical therapy services. Significant level and slope changes were detected using Poisson, beta, and linear regression models.

**Results:**

The March to September 2020 period was associated with significantly fewer home care admissions, discharges, and standardised assessments. Among those assessed with the interRAI Home Care assessment, significantly fewer patients received any personal support services. Among those assessed with either interRAI assessment and identified to have rehabilitation needs, significantly fewer patients received any therapy services. Among patients receiving services, patients received significantly fewer hours of personal support and fewer therapy visits per month. By September 2020, the rate of admissions and services had mostly returned to pre-pandemic levels, but completion of standardised assessments lagged behind.

**Conclusion:**

The first wave of the COVID-19 pandemic was associated with substantial changes in Ontario’s publicly funded home care system. Although it may have been necessary to prioritise service delivery during a crisis situation, standardised assessments are needed to support individualised patient care and system-level monitoring. Given the potential disruptions to home care services, future studies should examine the impact of the pandemic on the health and well-being of home care recipients and their caregiving networks.

## Introduction

On March 11, 2020, the World Health Organization declared the coronavirus disease caused by the SARS-CoV-2 virus (COVID-19) a pandemic. At the time, there were approximately 100 COVID-19 cases in Canada that were mostly linked to travel [1]. By September 30, 2020, Canada had recorded 158,758 COVID-19 cases and 9,327 COVID-19 deaths [2]. The effects of COVID-19 were disproportionately borne by residents and staff in long-term care settings, particularly in Quebec and Ontario. Through the first wave, 12% of COVID-19 cases and 75% of COVID-19 deaths in Canada occurred in long-term care homes [3]. Essential care partners and health system leaders identified a number of contributing factors in the long-term care system [4], many of which also applied to the home care system such as the lack of funding stability [5], increased patient acuity [5, 6], and a personal support workforce that is marginalised and under-valued [7]. Yet little is known about how the COVID-19 pandemic affected Canadians receiving publicly funded home care services.

Home care services refer to an array of home-based personal and professional supports, including but not limited to, personal support and homemaking services, nursing services, occupational therapy, physical therapy, and social work. Receiving care at home promotes independence and physical, mental, and social well-being while providing a less expensive alternative to institutional care and creating health system capacity [8, 9]. Individuals often rely on support from informal or unpaid caregivers such as family members. Many also receive care from formal providers who may be paid by the Ontario Health Insurance Plan (i.e., public insurance), private insurance, or out-of-pocket.

In Canada’s most populous province, an estimated 5.2% of Ontarians receive publicly insured home care services [8] that are coordinated by the 14 Home and Community Care Support Services organisations (HCCSS) and delivered by contracted service provider agencies. Once an individual is connected to their local HCCSS (either by referral or calling their organisation directly), HCCSS care coordinators assess the individual’s needs and develop the care plan. The interRAI Home Care assessment and the interRAI Contact Assessment are standardised assessments used with public home care patients in most Canadian provinces including Ontario. At the person level, standardised clinical assessments are used to identify the type and degree of needs, tailor care plans, and track health status. Organisations regularly submit interRAI assessment data to the Canadian Institute for Health Information (CIHI) for system-level monitoring of health outcomes and quality indicators.

interRAI is an international not-for-profit network of researchers and health and social service professionals who develop and support standardised comprehensive assessment tools and applications for a variety of health care settings [10–12]. The interRAI Home Care assessment (or its earlier version RAI-Home Care) and interRAI Contact Assessment have been used in Ontario’s publicly funded home care system since 2002 and 2010, respectively. Previous research has established the validity and reliability of these assessments [13–19]. The interRAI Contact Assessment (about 50 items) is used to screen new home care patients for key health and social needs and serves as a minimum data set for those who do not require further assessment. The interRAI Home Care assessment (about 250 items) is much more comprehensive and measures cognition, communication, mood and behaviour, psychosocial well-being, physical functioning, continence, disease diagnoses, health conditions, oral and nutritional status, skin condition, medication, treatments and procedures, social supports, environmental assessment, and discharge potential. The interRAI Home Care assessment also produces clinical scales and care planning protocols. In Ontario, the vast majority of new home care patients receive the interRAI Contact Assessment within 2 to 6 weeks, followed by an interRAI Home Care assessment if they are expected to require home care services for longer than two months (i.e., long-stay patients) [20]. Reassessments are normally done by regulated health professionals (e.g., nurses) every 6 to 12 months, or sooner if prompted by a significant change in the patient’s health.

Between March and June 2020, a CIHI report found that home care patients in four Canadian provinces were less likely to receive a standardised clinical assessment [21]. The CIHI report was unable to examine whether service patterns changed although other publications suggested a reduction in supply and demand for formal home care services during this period. Service provider agencies faced staffing shortages, individual providers experienced safety concerns and other job challenges, and patients and families may have placed their services on hold to limit the risk of viral transmission [22–25].

Changes in routine assessment and service provision could have led to individual- and system-level consequences. Missed assessments may have increased the risk of overlooking important health changes while missed services may have led to gaps in care and increased the burden on patients and families. We are aware that some jurisdictions completed non-standardised paper-based instruments early in the pandemic as a brief screening approach. This was concerning because these data were neither available to CIHI for its health system performance reports nor could be compared with standardised assessments completed in other health care settings (e.g., long-term care). Also, these instruments did not provide decision support tools (e.g., scale scores, risk algorithms) that could inform timely decision-making. For this reason, we focused on standardised assessments, which was distinct from the total number of assessments or assessed patients.

Our study sought to compare the patterns in home care episode, standardised assessment, and service volumes in Ontario’s publicly funded home care system before and during the COVID-19 pandemic. The goal was to provide an understanding of the province’s home care operations during the pandemic and help to inform future strategies to ensure continuity of assessment approaches in the face of system-level crises.

## Materials and methods

### Study design and setting

We plotted monthly time series data for publicly funded home care recipients in Ontario, Canada. Data from March 2020 to September 2020 represented the period of interest (i.e., during the COVID-19 pandemic) and data from March 2019 to February 2020 were used for comparison (i.e., before the COVID-19 pandemic).

### Data sources

Ontario Health maintains the Home Care Database (HCD) that stores assessment and administrative data on all publicly funded home care services coordinated by HCCSS and delivered and paid to service provider agencies. An existing data sharing agreement permitted the transfer of data from Ontario Health to the interRAI Canada research group at the University of Waterloo. All data were anonymised by Ontario Health although a linking field (i.e., patient identifier) was generated to allowing merging of data tables. Use of these data and the processes in place to protect patient privacy and confidentiality were approved by the University of Waterloo’s Office of Research Ethics (ORE# 18228). At the time of writing, data up to September 2020 were available.

#### Assessment data

This study used the following validated scales and algorithms from the interRAI Home Care assessment: Activities of Daily Living Hierarchy Scale (ADLH) ranges from 0 to 6 with higher levels indicating greater difficulty in performing activities of daily living [26]; Cognitive Performance Scale 2 (CPS2) ranges from 0 to 8 with higher levels indicating greater cognitive impairment [27]; Depression Rating Scale (DRS) ranges from 0 to 14 with higher levels indicating more and/or frequent depressive symptoms [28]; Communication Scale ranges from 0 to 8 with higher levels indicating greater difficulty with making self-understood and ability to understand others; Changes in Health, End-stage disease, Signs, and Symptoms Scale (CHESS) ranges from 0 to 5 with higher levels indicating greater health instability [29, 30]; Personal Support (PS) Algorithm ranges from 1 to 6 with higher groups suggesting greater need for personal support services [31]. Additionally, items assessing recent changes in decision-making and functional status were used to code for cognitive and functional decline. This study also used the following from the interRAI Contact Assessment: CHESS-CA ranges from 0 to 5 and has the same interpretation as CHESS with higher levels indicating greater health instability [32]; and Rehabilitation Algorithm ranges from 1 to 5 with higher levels suggesting greater need for therapy services [33].

#### Administrative data

Patient-level demographic information, admission and discharge dates, and home care services were retrieved from HCD. Personal support services and shift nursing were reported in the number of hours. Non-shift nursing, occupational therapy, and physical therapy were reported in the number of visits. In this paper, nursing hours and visits were summed to represent total nursing services.

### Sample

The full sample comprised of all adults (age ≥ 18 years) who received publicly funded home care services in Ontario between March 2019 and September 2020. The full sample was used to report on monthly admissions and discharges, interRAI Home Care assessments, interRAI Contact Assessments, and home care services. We created a sub-sample by linking each patient’s services with their most recent assessment, which was used to stratify service patterns by indicators of potential need. For interRAI Home Care assessments that are typically done within 12 months, we applied a 13-month lookback period up to February 2020 (i.e., before the COVID-19 pandemic). We extended the lookback period to 16 months between March and September 2020 to allow for overdue assessments during the COVID-19 pandemic. For interRAI Contact Assessments that are typically done within 6 weeks, we applied a 2-month lookback period. Services that could not be linked to any standardised assessment were excluded. This sub-sample was used to report on the receipt of personal support services by PS Algorithm, nursing services by CHESS and CHESS-CA, and occupational and physical therapy services by cognitive or functional decline and Rehabilitation Algorithm. Details about the sample selection were summarised in a flow diagram provided in the S1 Fig. To aid with readability, we used “screening assessment” and “comprehensive assessment” in place of the official instrument names in the results and discussion sections.

### Analysis

For each calendar month, total counts were presented using line and bar charts. We performed interrupted time series analyses using Poisson, beta, and linear regression models to detect significant changes in trends [34]:

$$Outcome_{t}=\;\beta_{0}+\;\beta_{1}Time+\;\beta_{2}COVID_{t}+\;\beta_{3}Time\times COVID_{t}$$

where β_1_ is the slope during the pre-pandemic period (i.e., March 2019 to February 2020) and β_2_ and β_3_ are the level and slope changes during the pandemic period (i.e., March 2020 to September 2020), respectively. For patients with a linkable interRAI assessment, we examined the receipt of home care services in two ways: the proportion of patients receiving any home visit of the service type, and the adjusted monthly number of visits (or hours) among those receiving any home visit of the service type. We adjusted the amount of services by the number of valid days, so that time before a patient was admitted or after a patient was discharged was not counted in the denominator. To account for lagged effects, the service models were run with both March and April 2020 as the start of the pandemic period. Chi-Square tests were used to detect significant differences in patient characteristics before and after the pandemic period. We selected a significance threshold of p<0.05. All analyses were done using SAS 9.4 (SAS Institute Inc., Cary, NC).

## Results

**Fig 1:**
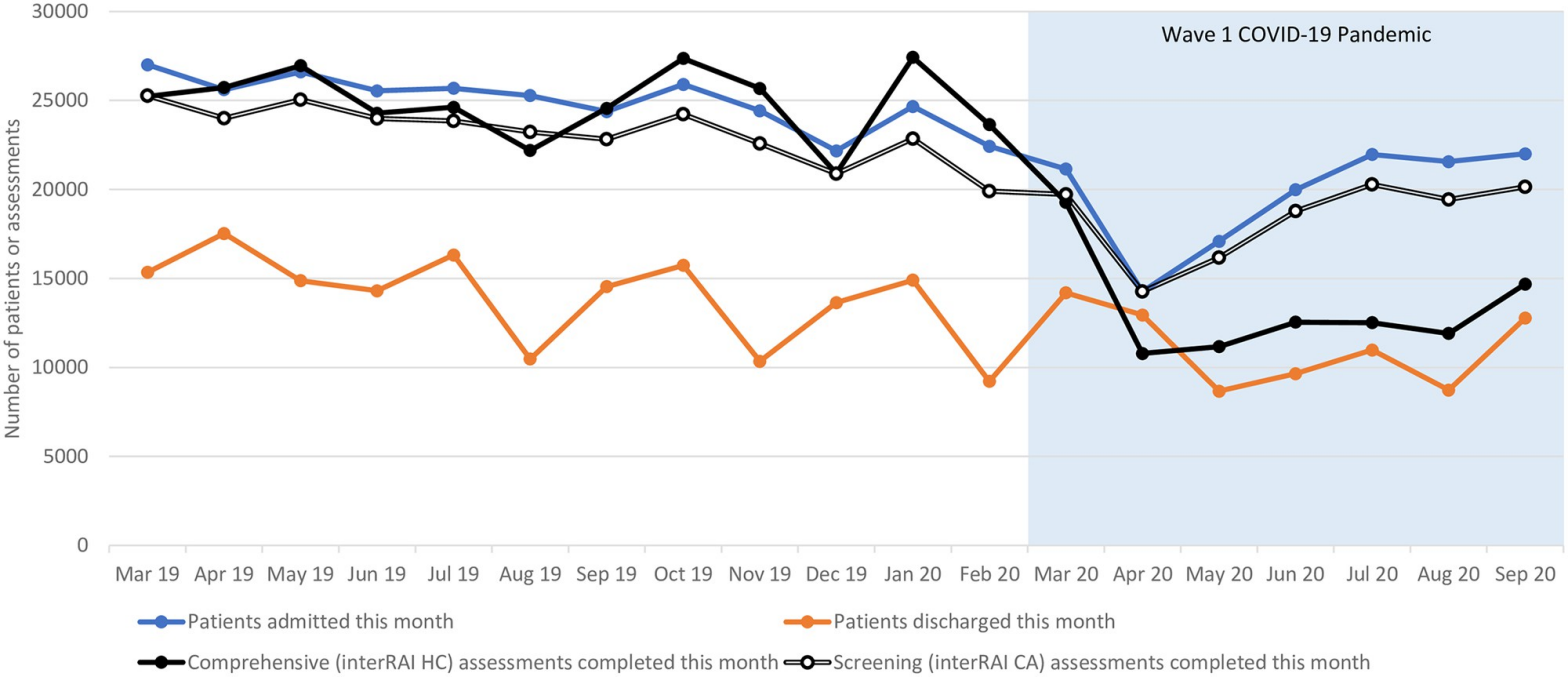
Home care admissions, discharges, and assessments.

Before the COVID-19 pandemic, Ontario’s publicly funded home care system admitted 31,105 patients and discharged 30,625 patients in an average month (Fig 1). At the onset of the pandemic, admissions fell by 10.2% and 37.8% in March and April, respectively (β_2_: p<0.0001). In contrast, discharges increased by 4.0% in March then fell by 16.6% and 32.0% in April and May, respectively (β_2_: p<0.0001). During the ensuing months, both volumes increased until the number of admissions and discharges had reached 97% and 95% of their pre-pandemic averages, respectively (β_3_: p<0.0001).

Before the COVID-19 pandemic, Ontario’s publicly funded home care system completed 22,741 comprehensive assessments and 23,557 screening assessments in an average month (Fig 1). There was a slight but significant downward trend in assessment volumes from March 2019 to February 2020 (β_1_: p<0.0001). At the onset of the pandemic, comprehensive assessments declined by 25.6% and 57.7% in March and April, respectively (β_2_: p<0.0001). Screening assessments followed a less steep decline in the same period, where the number of screening assessments fell by 15.7% and 38.5% (β_2_: p<0.0001). Although both assessments demonstrated a significant positive trend during the pandemic period (β_3_: p<0.0001), volumes remained lower than usual. By September 2020, the number of comprehensive and screening assessments had reached 59% and 88% of their pre-pandemic averages, respectively. As expected, the pattern of screening assessments (that are typically used to assess new home care patients) appeared the mirror the pattern of admissions.

Table 1 compared sociodemographic and clinical characteristics of home care patients who received a comprehensive assessment between March and September in 2019 and 2020 (as depicted by the black line in Fig 1). While the proportions of assessed patients older than 65 years and identifying as female did not vary significantly between cohorts, there were small but significant differences in marital status, living arrangement, and residence type. Post-hoc comparisons showed that significantly more long-stay patients assessed during the COVID-19 period had never been married, lived alone or lived with relatives other than a spouse/partner, and lived in a private home/apartment or rented room. Long-stay patients assessed during the COVID-19 period also had significantly more complex health needs across the major clinical domains. The prevalence of health instability increased from 27.0% to 31.5%, communication impairment increased from 16.1% to 17.8%, and cognitive impairment increased from 43.9% to 47.4%.

**Table 1:** Comparison of sociodemographic and clinical characteristics of home care patients receiving a comprehensive assessment from March to September, in 2019 and 2020.

% (n)		Mar–Sep 2019 (n = 159,023)	Mar–Sep 2020 (n = 83,145)	p-value
**Sociodemographic characteristics**				
Age	65+ years	86.5 (137,494)	86.3 (71,787)	0.40
Sex	Female	61.9 (98,434)	61.8 (51,410)	0.75
Marital status	Never married	9.4 (14,886)	10.1 (8,361)	<0.001
	Married or have partner/significant other	38.4 (61,128)	37.7 (31,310)	
	Widowed	41.8 (66,466)	41.2 (34,244)	
	Separated or divorced	10.4 (16,543)	11.1 (9,230)	
Living arrangement	Lives alone	32.7 (52,053)	33.3 (27,720)	<0.001
	Lives with spouse or partner (with or without other relatives)	35.4 (56,245)	34.5 (28,693)	
	Lives with other relatives (not with spouse or partner)	19.7 (31,316)	20.5 (17,028)	
	Lives with non-relatives	12.2 (19,409)	11.7 (9,704)	
Residence type	Private home/apartment or rented room	82.9 (131,839)	84.5 (70,244)	<0.001
	Assisted living or semi-independent living	12.8 (20,274)	11.4 (9,436)	
	Other	4.4 (6,910)	4.2 (3,465)	
**Clinical characteristics**				
Functional status	Moderate to severe ADL impairment^a^	35.5 (56,512)	38.1 (31,671)	<0.001
Cognitive status	Moderate to severe cognitive impairment^b^	43.9 (69,778)	47.4 (39,422)	<0.001
Mood symptoms	Presence of symptoms of moderate to severe depression^c^	21.4 (34,020)	22.2 (18,430)	<0.001
Communication: expression and comprehension	Moderate to severe communication impairment^d^	16.1 (25,557)	17.8 (14,818)	<0.001
Health instability	Moderate to very high health instability^e^	27.0 (42,930)	31.5 (26,176)	<0.001

^a^ Activities of Daily Living Hierarchy Scale (ADLH) ≥ 3.

^b^ Cognitive Performance Scale 2 (CPS2) ≥ 4.

^c^ Depression Rating Scale (DRS) ≥ 3.

^d^ Communication Scale ≥ 4.

^e^ Changes in Health, End-stage disease, and Signs and Symptoms Scale (CHESS) ≥ 3.

**Fig 2:**
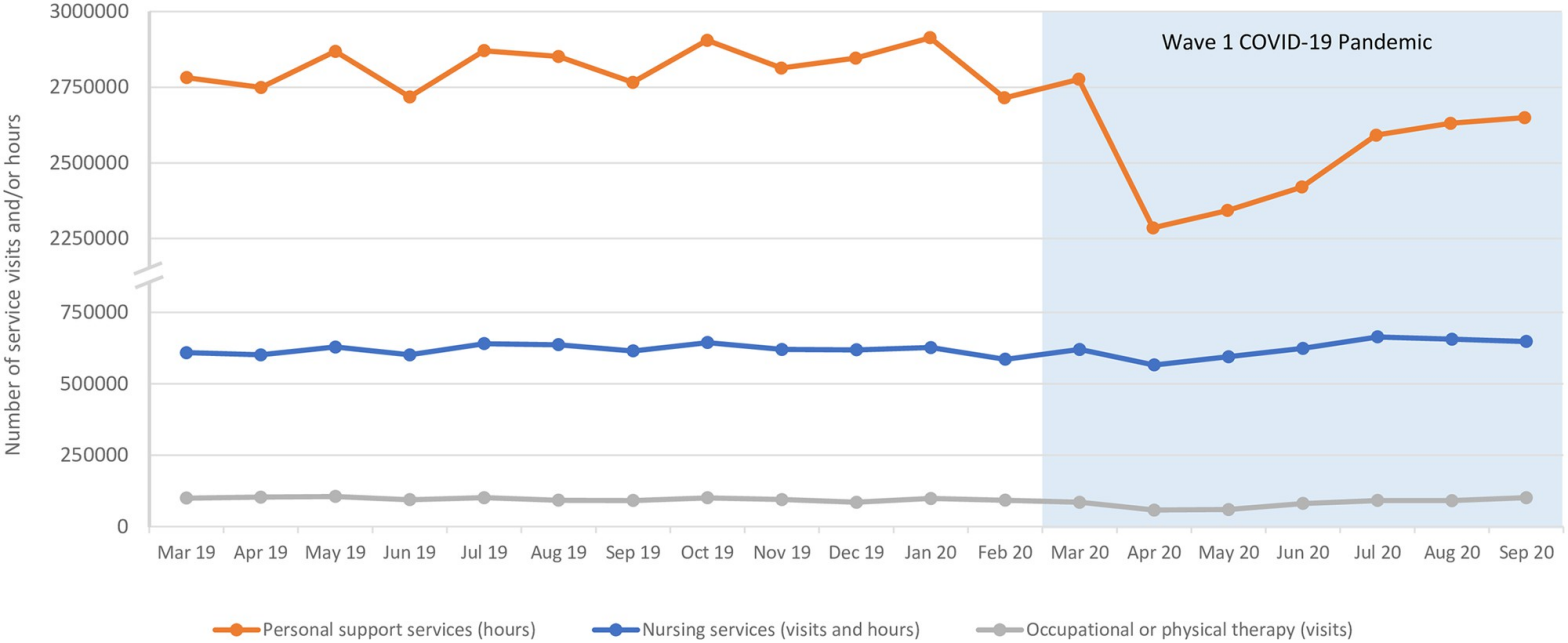
Home care service visits and/or hours, by service type.

Before the COVID-19 pandemic, Ontario’s publicly funded home care system coordinated the delivery of approximately 2.9 million hours of personal support services, 600,000 units of nursing services (combined hours and visits), and nearly 100,000 therapy visits in a typical month (Fig 2). Personal support services fell by 1.4% and 18.9% in March and April, respectively. Nursing services increased by 0.2% in March and fell by 8.5% in April. Occupational and physical therapy services declined by 11.9% and 40.2% in March and April, respectively. All service types reached their lowest volumes in April and increased steadily during the ensuing months. By September 2020, the volumes of personal support, nursing, and therapy services had reached 94%, 105%, and 105% of the pre-pandemic averages, respectively.

**Fig 3:**
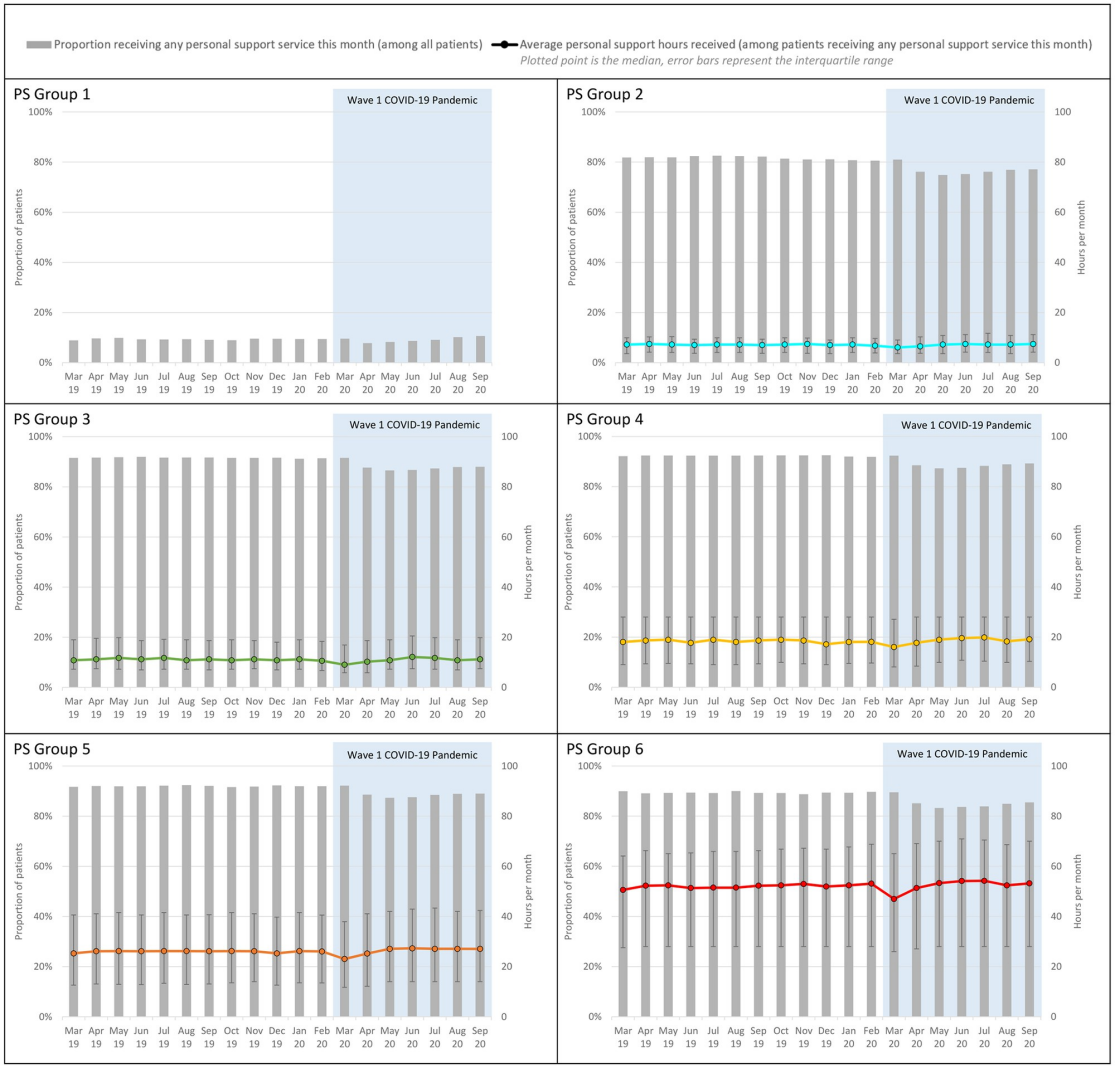
Receipt of personal support services among patients assessed with comprehensive assessment, by personal support algorithm.

Fig 3 examined the receipt of personal support services among long-stay patients stratified by increasing need for personal support services defined by higher PS Algorithm groups. Among those assessed with the comprehensive assessment, patients in all PS Groups were significantly less likely to receive any personal support services in either March or April 2020 (all β_2_: p<0.05). The relative declines were similar across groups, averaging about -3% to -5% from their pre-pandemic averages. For those receiving any personal support services, the onset of the pandemic was also associated with lower median allocations (all β_2_: p<0.05). In March 2020, the adjusted monthly amount of personal support ranged from -0.8 hours in PS Group 2 (-11.5% change) to -2.9 hours in PS Group 6 (-5.6% change) compared to the previous year. Across most PS groups, the interaction terms representing the slopes during the pandemic period were positive and significant, indicating both the likelihood of receiving personal support services and the amount of personal support services received increased over time. By September 2020, median allocations exceeded pre-pandemic averages by 4 to 6%.

**Fig 4:**
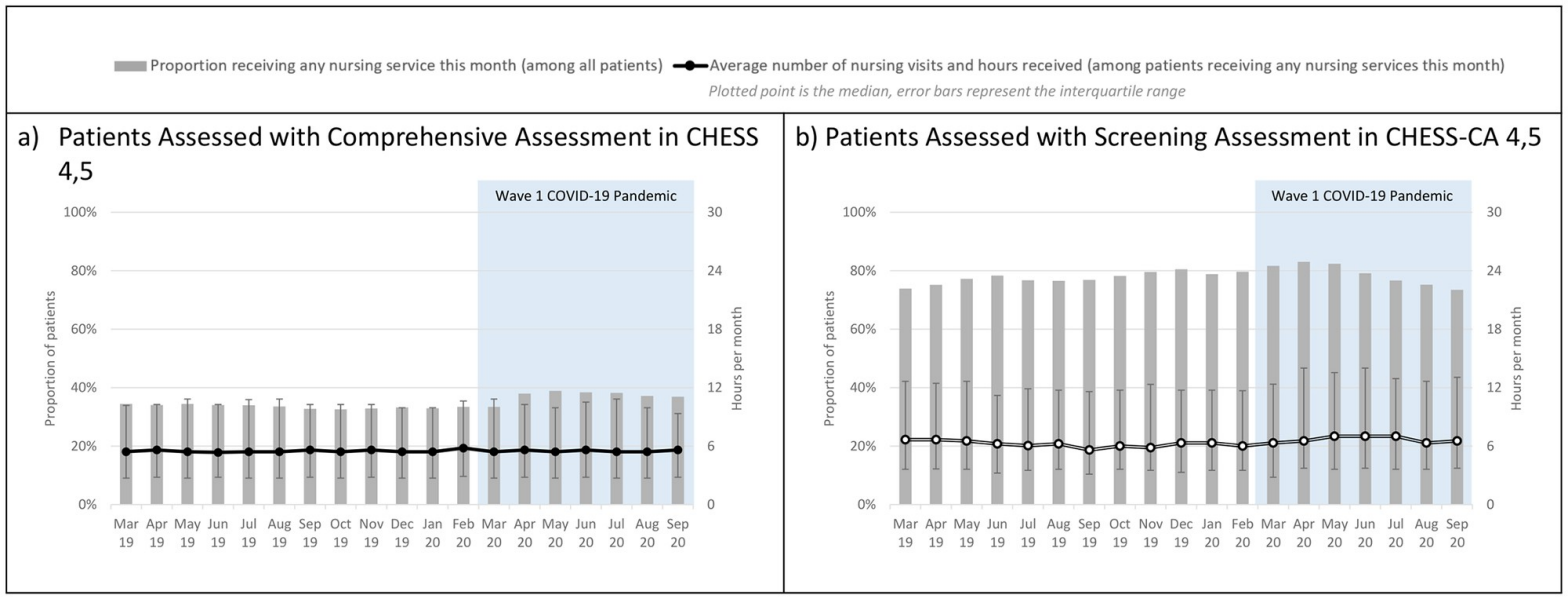
Receipt of nursing services (including shift nursing) among patients with high health instability.

Fig 4 examined the receipt of nursing services among patients identified to have high levels of health instability (i.e., CHESS or CHESS-CA 4 or 5). Among those assessed with the comprehensive assessment, the proportion of patients with high health instability who received any nursing services significantly increased from 33.5% (pre-pandemic average) to 37.9% (April 2020) (β_2_: p = 0.001). Among those assessed with the screening assessment, the proportion of patients with high health instability who received any nursing services significantly increased from 77.6% (pre-pandemic average) to 83.0% (April 2020) (β_2_: p = 0.03). The interaction terms were negative and significant, indicating these proportions fell in the subsequent months (β_2_: p<0.05). No significant level or slope changes were observed in the adjusted monthly amount of nursing services.

**Fig 5:**
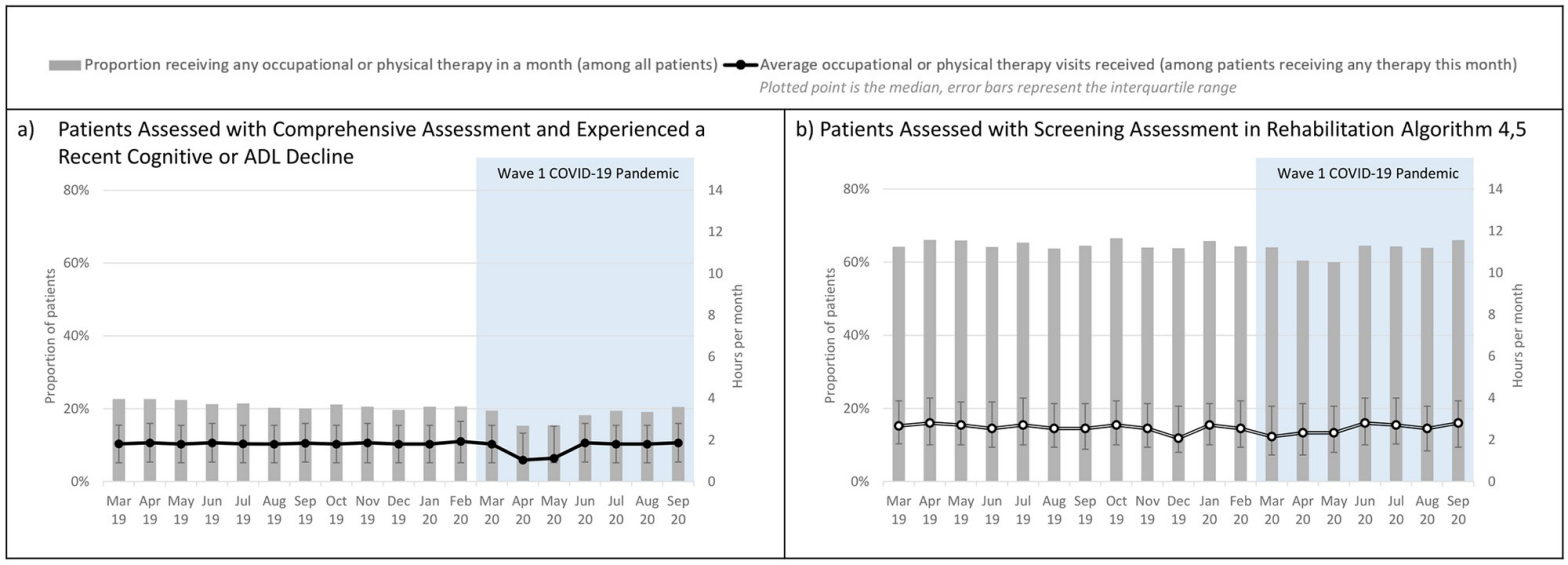
Receipt of occupational or physical therapy among patients with potential rehabilitation needs.

Fig 5 examined the receipt of occupational or physical therapy services among patients with potential rehabilitation needs. Among those assessed with the comprehensive assessment, the proportion of patients who experienced a recent decline in cognitive or functional status and received any therapy services fell from 21.1% (pre-pandemic average) to 15.4% (April 2020) (β_2_: p<0.0001). The median amount of therapy services also fell from 1.8 hours per month (pre-pandemic average) to 1.0 hours per month (April 2020) (β_2_: p<0.0001). A similar pattern of decline was observed for patients assessed with the screening assessment and identified to have high rehabilitation needs, where the proportion receiving any therapy services fell from 64.9% (pre-pandemic average) to 60.4% (April 2020) (β_2_: p = 0.01). The median amount of therapy services also fell from 2.6 hours per month (pre-pandemic average) to 2.3 hours per month (April 2020) (β_2_: p<0.0001). All interaction terms representing the slopes during the pandemic period were positive and significant. By September 2020, the proportion of long-stay and short-stay patients receiving any therapy services had reached 97% and 102% of the pre-pandemic averages, respectively.

## Discussion

Ontario was substantially affected by the first wave of the COVID-19 pandemic. By September 2020, Ontario recorded 51,710 COVID-19 cases and 2,848 COVID-19 deaths, accounting for 32.6% and 30.6% of Canada’s COVID-19 cases and deaths [2]. Numerous reports highlighted the wide-ranging impacts of the pandemic across the health system, including substantial outbreaks and extended lockdowns in long-term care homes [35, 36], fewer preventive and chronic care visits in primary care settings [37], as well as fewer emergency department visits and hospital presentations and cancellations of planned surgeries [38–40]. Our findings were consistent with a recent CIHI report [21] that Ontario’s publicly funded home care system completed significantly fewer standardised assessments during the March to September 2020 period. Further, our study demonstrated that this period was significantly associated with fewer home care admissions and discharges, and reductions in both the proportion of patients receiving personal support and therapy and the amount of these services received per patient. By September 2020, the rate of admissions and services had mostly returned to pre-pandemic levels; however, the recovery of standardised assessments lagged behind.

Comprehensive assessments were more affected than screening assessments, which can at least be partially explained by differences in target populations and instrument design. The screening assessment is typically used at home care intake; therefore, it was expected that the volume of screening assessments recovered as quickly as home care admissions. As well, the screening assessment was validated for both in-person and phone use, which made it easier for assessors to pivot within existing practice to phone assessments. Accordingly, CIHI reported that there was a 53% increase in phone-based screening assessments between April and June 2020 compared to the same period in 2019 [21]. In contrast, the comprehensive assessment was designed to be completed in-person in the patient’s home, so that assessors integrate visual/sensory information about the patient and their home environment in addition to the patient and family’s reported needs. Although interRAI released a guideline for completing the comprehensive assessment via video conferencing in March 2020 [41], patients may have had difficulties setting up or using the technology (e.g., positioning the camera) and providers would have needed time to update assessment policies and build confidence in the quality of data.

Among those receiving a standardised assessment, the pandemic appeared to change patterns of personal support and therapy services moreso than nursing services. Therapy services declined by the largest percentage, raising the question of whether rehabilitation services were more likely to be perceived as care that could be delayed. Jones and colleagues [42] observed the same phenomenon among home care recipients with dementia and hypothesised that nursing services may have been considered more essential than other home care services. Where rehabilitation promotes functional reserve and can reduce the risk of poor outcomes such as falls, frailty, and hospitalisation, we argue that therapy services serve a critical role, particularly during a time of limited health system capacity [43]. Rapid adoption of virtual rehabilitation was likely a major contributor to recovery, for which there is emerging evidence of high quality of care and patient satisfaction [44].

We also found that fewer patients received personal support services and patients received fewer hours of services, which was consistent with the experiences of home care patients and their caregivers reported in other studies [23, 24]. In this study, we further demonstrated that these patterns held true regardless of the degree of help needed with personal care. We speculate this reflected the similar opportunities and challenges created by the pandemic. For some, remote work or school arrangements offered more flexibility and time at home to be able to offer more caregiving, thus reducing the need for paid services [22, 45]. Other families may have opted to cancel or pause services to mitigate the risk of virus transmission, especially in cases where multiple service providers were involved or service providers also worked in long-term care homes [23, 24]. At the same time, caregiving networks may not have been able to completely replace the personal care delivered by formal providers. Many caregivers took on more caregiving responsibilities amid other commitments such as work, school, or childcare [45]. Additionally, families had to balance different safety risks when deciding whether to continue home care services. Although many caregivers reported high levels of anxiety related to exposure risks, they also feared they would not be able to manage without additional help, which would place both the care recipient and caregiver at risk of worsening health [24, 45, 46]. Families may have negotiated these risks by reducing the number of visits or limiting the number of service providers in the home.

Although this study focused on paid home care services, it is important to understand the impacts of service changes on unpaid caregiving. Even before the appearance of COVID-19, one in three unpaid caregivers of home care patients experienced caregiver distress [47]. During the pandemic, unpaid caregivers had fewer options to ask for or hire external help despite being in even greater need of respite [45]. Distressed caregivers have been typically represented by those caring for loved ones with substantial personal care needs [47], but these analyses raise the question of whether home care service disruptions (particularly of personal support services) may have affected a broader group of caregivers. The sustainability of community-based care relies on protecting the health and well-being of caregivers.

This study has important implications for home care practice, research, and quality monitoring. Although Ontario’s publicly funded home care system appeared to be functioning closer to normal, comprehensive assessment volumes remained 41% lower in September 2020 compared to the previous year. From a practice standpoint, this meant a substantial proportion of the home care population was not being (re-)assessed with a standardised assessment. During the first wave, some jurisdictions allowed assessors to complete the interRAI Home Care assessment by phone, while others continued to mandate in-person visits. Other jurisdictions switched to the interRAI Contact Assessment for patients for whom they would normally have completed a comprehensive assessment. As the province entered the second wave, some jurisdictions adopted the interRAI Check-Up Self-Report assessment intended to be used with patients with lighter care needs [48]. The alternatives, either postponing standardised assessments or adopting non-standardised assessments, were unsustainable because they could not provide a full picture of which patients may have gotten worse (with or without a positive COVID-19 status) and the magnitude of the problem. Importantly, home care clinicians and administrators should re-establish standardised assessments as a key function of home care operations. It is important to not lose sight of patient needs in the midst of any major crisis, including a global pandemic, and it is also essential to identify changes in individual health that have ongoing consequences (e.g., new mental health concerns, functional decline, exacerbations of chronic disease).

Research and quality monitoring in home care is also enabled by standardised assessments. For instance, some of this paper’s co-authors used RAI-MDS 2.0 data to compare the prevalence of resident depression, delirium, and behaviour problems and measure the effect of COVID-19 lockdowns in long-term care homes [49]. Likewise, standardised data in the home care sector can be used to measure the impact of the pandemic on patient health and well-being. In this study, home care patients who were assessed during the pandemic had worse health instability, communication impairment, and cognitive impairment compared to the previous year, but future studies should discern whether this was due to HCCSS prioritising standardised assessments for the most complex patients or whether this represented a real change in the health status of the home care population. In this paper, we highlighted the importance of caregiver well-being and strongly recommend that the sector utilise the caregiving questions already embedded within interRAI assessments to screen for individual caregiver needs as well as monitor levels of caregiver distress across the system. To pursue these studies, researchers will need to account for missing standardised assessments during the height of the pandemic. Initial and routine assessments completed during this time captured a slightly more complex population and were not representative of the whole home care population. Researchers will also need to consider adapting observation periods to account for overdue assessments. It may also be useful to analyse home care outcomes at the regional level since individual HCCSS may have adapted their assessment policies in different ways. By choosing methodological designs thoughtfully and through careful interpretation, we believe that information gained from home care assessments (despite the reduced volumes) can be used to support quality monitoring and policymaking.

Since 2002, the use of interRAI assessments across Ontario home care has enabled census-level research and monitoring of the home care sector. However, as this paper demonstrates, the quality and completeness of the data relies on administrators and assessors to maintain the assessment standard, even during times of crisis. For readers, we highlight the following limitations of our analyses. First, this study used open-year data that CIHI defines as data received before the official annual submission deadline, which may change or be partially complete [21]. Second, this study applied a short list of exclusions such as linking referrals and assessments within a given timeframe that did not exactly match the CIHI methodology. Nevertheless, the changes reported in this study did not deviate beyond 1 or 2 percentage points of the CIHI report and overall conclusions did not change [21]. Third, when interpreting the sub-sample results, it is important to note that a patient’s most recent interRAI assessment may not have been an accurate reflection of their health status at the time of service delivery, especially if they had missed or delayed assessments due to the COVID-19 pandemic. Fourth, the sub-sample results are applicable only to patients who received a standardised assessment; thus, these results should not be extrapolated to newly admitted or existing patients who did not receive a standardised assessment. Fifth, our findings were based on province-wide data that may not necessarily apply to individual HCCSS due to differences in assessment practices. HCCSS organisations that had a quicker recovery in the use of standardised assessments would be over-represented in the sub-sample. Sixth, we neither had access nor the means to analyse non-standardised assessment data that would have helped answer the question of whether apparent changes in acuity represented sampling bias or meaningful changes in health status (i.e., Table 1). Lastly, we did not have access to data on whether or when a patient’s services were put on hold, so we could not exclude on-hold or waitlisted days to calculate service utilisation.

## Conclusion

Across Ontario’s publicly funded home care system, the first wave of the COVID-19 pandemic significantly disrupted patterns of home care admissions, discharges, standardised assessments, as well as receipt of personal support, occupational therapy, and physical therapy services. While the home care sector demonstrated its ability to pivot quickly and reverse many of these trends, service disruptions coupled with a pullback in standardised assessments placed home care patients at risk. These risks included placing the responsibility of bridging the care gap on patients and families and not adequately prioritising standardised assessments that are necessary for individual- and system-level monitoring. We conclude that the sector should prioritise both home care assessment and service delivery during a crisis to ensure persons who rely on these essential services are well-supported in the community.

## Supporting information

S1 Fig Sample flow diagram. (TIFF)

S1 Table Parameter estimates from regression models. (DOCX)
