# Supplementary figures and images for: Patterns of home care assessment and service provision before and during the COVID-19 pandemic in Ontario, Canada
Source: PLoS One. 2022 Mar 30;17(3):e0266160. doi: 10.1371/journal.pone.0266160 (PMC8966998; doi:10.1371/journal.pone.0266160)

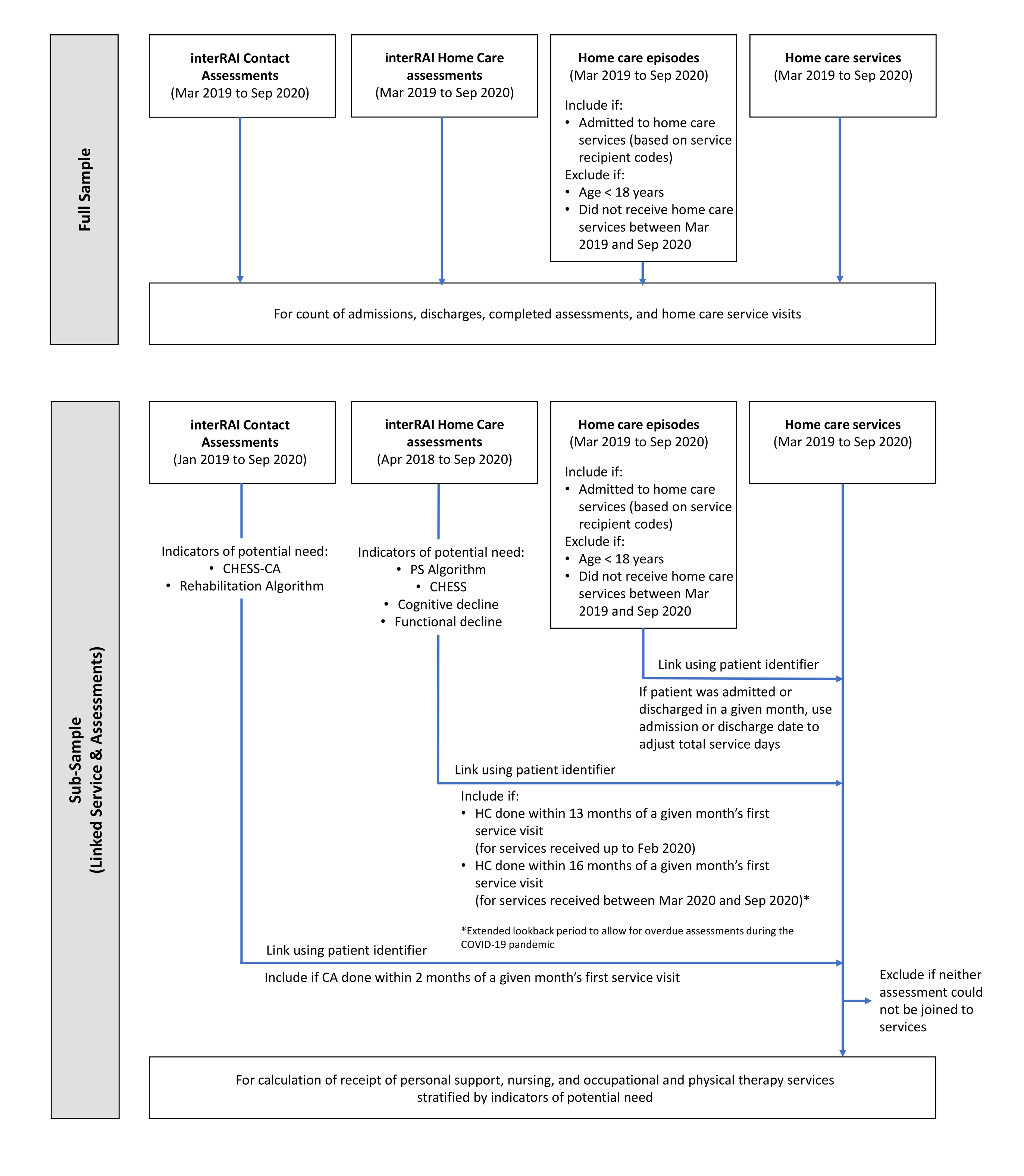

Supplement: S1 Fig — (TIFF) [file pone.0266160.s001.tiff]
