# Supplementary material for: Patterns of home care assessment and service provision before and during the COVID-19 pandemic in Ontario, Canada
Source: PLoS One. 2022 Mar 30;17(3):e0266160. doi: 10.1371/journal.pone.0266160 (PMC8966998; doi:10.1371/journal.pone.0266160)
Supplement: S1 Table — (DOCX) [file pone.0266160.s002.docx]

**Supplementary Tables**

Parameter Estimates for Count of Admissions and Discharges from Poisson Regression Models

|  | Admissions | | | Discharges | | |
| --- | --- | --- | --- | --- | --- | --- |
|  | Estimate | Standard error | p-value | Estimate | Standard error | p-value |
| β_2_ (pandemic-associated change in level) | -0.926 | 0.019 | <0.001 | -0.130 | 0.019 | <0.001 |
| β_3_ (pandemic-associated change in slope) | 0.051 | 0.001 | <0.001 | -0.006 | 0.001 | <0.001 |

Parameter Estimates for Count of Standardised Assessments from Poisson Regression Models

|  | Comprehensive assessments | | | Screening assessments | | |
| --- | --- | --- | --- | --- | --- | --- |
|  | Estimate | Standard error | p-value | Estimate | Standard error | p-value |
| β_2_ (pandemic-associated change in level) | -0.873 | 0.023 | <0.001 | -0.3274 | 0.028 | <0.001 |
| β_3_ (pandemic-associated change in slope) | -0.017 | 0.002 | <0.001 | 0.050 | 0.002 | <0.001 |

Parameter Estimates for Proportion Receiving Any Personal Support Services from Beta Regression Models – All PS Groups (March 2020)

|  | β_2_ (pandemic-associated change in level) | | | β_3_ (pandemic-associated change in slope) | | |
| --- | --- | --- | --- | --- | --- | --- |
| PS Group | Estimate | Standard error | p-value | Estimate | Standard error | p-value |
| 1 | -0.524 | 0.197 | 0.01 | 0.034 | 0.013 | 0.001 |
| 2 | -0.143 | 0.105 | 0.17 | -0.001 | 0.003 | 0.85 |
| 3 | 0.089 | 0.146 | 0.54 | -0.020 | 0.010 | 0.05 |
| 4 | -0.216 | 0.178 | 0.23 | -0.006 | 0.012 | 0.61 |
| 5 | 0.072 | 0.177 | 0.68 | -0.017 | 0.012 | 0.17 |
| 6 | -0.039 | 0.163 | 0.81 | -0.012 | 0.011 | 0.27 |

Parameter Estimates for Proportion Receiving Any Personal Support Services from Beta Regression Models – All PS Groups (April 2020)

|  | β_2_ (pandemic-associated change in level) | | | β_3_ (pandemic-associated change in slope) | | |
| --- | --- | --- | --- | --- | --- | --- |
| PS Group | Estimate | Standard error | p-value | Estimate | Standard error | p-value |
| 1 | -1.127 | 0.337 | 0.001 | 0.070 | 0.020 | 0.001 |
| 2 | -1.038 | 0.174 | <0.001 | 0.055 | 0.010 | <0.001 |
| 3 | -1.016 | 0.241 | <0.001 | 0.048 | 0.014 | 0.001 |
| 4 | -1.572 | 0.301 | <0.001 | 0.081 | 0.018 | <0.001 |
| 5 | -0.980 | 0.296 | 0.001 | 0.048 | 0.018 | 0.008 |
| 6 | -1.059 | 0.276 | <0.001 | 0.052 | 0.017 | 0.002 |

Parameter Estimates for Adjusted Monthly Amount of Personal Support from Linear Regression Models – All PS Groups (March 2020)

|  | β_2_ (pandemic-associated change in level) | | | β_3_ (pandemic-associated change in slope) | | |
| --- | --- | --- | --- | --- | --- | --- |
| PS Group | Estimate | Standard error | p-value | Estimate | Standard error | p-value |
| 1 | -- | -- | -- | -- | -- | -- |
| 2 | -2.470 | 0.376 | <0.001 | 0.164 | 0.025 | <0.001 |
| 3 | -2.828 | 0.565 | <0.001 | 0.180 | 0.037 | <0.001 |
| 4 | -4.152 | 1.150 | <0.001 | 0.273 | 0.077 | <0.001 |
| 5 | -2.694 | 1.207 | 0.02 | 0.138 | 0.080 | 0.09 |
| 6 | -9.448 | 2.367 | <0.001 | 0.588 | 0.157 | <0.001 |

Parameter Estimates for Adjusted Monthly Amount of Personal Support from Linear Regression Models – All PS Groups (April 2020)

|  | β_2_ (pandemic-associated change in level) | | | β_3_ (pandemic-associated change in slope) | | |
| --- | --- | --- | --- | --- | --- | --- |
| PS Group | Estimate | Standard error | p-value | Estimate | Standard error | p-value |
| 1 | -- | -- | -- | -- | -- | -- |
| 2 | 0.261 | 0.646 | 0.68 | 0.026 | 0.039 | 0.50 |
| 3 | 0.391 | 0.988 | 0.69 | 0.020 | 0.059 | 0.74 |
| 4 | 4.524 | 1.948 | 0.02 | -0.182 | 0.116 | 0.12 |
| 5 | 4.375 | 2.096 | 0.04 | -0.216 | 0.125 | 0.09 |
| 6 | 5.636 | 4.117 | 0.17 | -0.187 | 0.245 | 0.45 |

Parameter Estimates for Proportion of Receiving Any Nursing Services from Beta Regression Models – CHESS 4 or 5

|  | March 2020 | | | April 2020 | | |
| --- | --- | --- | --- | --- | --- | --- |
|  | Estimate | Standard error | p-value | Estimate | Standard error | p-value |
| β_2_ (pandemic-associated change in level) | -0.102 | 0.123 | 0.41 | 0.709 | 0.212 | 0.001 |
| β_3_ (pandemic-associated change in slope) | 0.020 | 0.008 | 0.02 | -0.032 | 0.013 | 0.01 |

Parameter Estimates for Adjusted Monthly Amount of Nursing Services from Linear Regression Models – CHESS 4 or 5

|  | March 2020 | | | April 2020 | | |
| --- | --- | --- | --- | --- | --- | --- |
|  | Estimate | Standard error | p-value | Estimate | Standard error | p-value |
| β_2_ (pandemic-associated change in level) | -0.620 | 0.743 | 0.04 | 0.678 | 1.293 | 0.60 |
| β_3_ (pandemic-associated change in slope) | 0.065 | 0.049 | 0.19 | -0.013 | 0.077 | 0.86 |

Parameter Estimates for Proportion of Receiving Any Nursing Services from Beta Regression Models – CHESS-CA 4 or 5

|  | March 2020 | | | April 2020 | | |
| --- | --- | --- | --- | --- | --- | --- |
|  | Estimate | Standard error | p-value | Estimate | Standard error | p-value |
| β_2_ (pandemic-associated change in level) | 0.684 | 0.352 | 0.05 | 1.313 | 0.608 | 0.03 |
| β_3_ (pandemic-associated change in slope) | -0.051 | 0.023 | 0.03 | -0.084 | 0.036 | 0.02 |

Parameter Estimates for Adjusted Monthly Amount of Nursing Services from Linear Regression Models – CHESS-CA 4 or 5

|  | March 2020 | | | April 2020 | | |
| --- | --- | --- | --- | --- | --- | --- |
|  | Estimate | Standard error | p-value | Estimate | Standard error | p-value |
| β_2_ (pandemic-associated change in level) | 0.219 | 0.912 | 0.81 | 1.969 | 1.589 | 0.21 |
| β_3_ (pandemic-associated change in slope) | 0.005 | 0.061 | 0.93 | -0.104 | 0.095 | 0.27 |

Parameter Estimates for Proportion of Receiving Any Therapy Services from Beta Regression Models – Recent Cognitive or ADL Decline

|  | March 2020 | | | April 2020 | | |
| --- | --- | --- | --- | --- | --- | --- |
|  | Estimate | Standard error | p-value | Estimate | Standard error | p-value |
| β_2_ (pandemic-associated change in level) | -0.904 | 0.144 | <0.001 | -1.482 | 0.248 | <0.001 |
| β_3_ (pandemic-associated change in slope) | 0.055 | 0.009 | <0.001 | 0.093 | 0.015 | <0.001 |

Parameter Estimates for Adjusted Monthly Amount of Therapy from Linear Regression Models – Recent Cognitive or ADL Decline

|  | March 2020 | | | April 2020 | | |
| --- | --- | --- | --- | --- | --- | --- |
|  | Estimate | Standard error | p-value | Estimate | Standard error | p-value |
| β_2_ (pandemic-associated change in level) | -0.994 | 0.262 | <0.001 | -1.94 | 0.463 | <0.001 |
| β_3_ (pandemic-associated change in slope) | 0.048 | 0.017 | 0.008 | 0.115 | 0.028 | <0.001 |

Parameter Estimates for Proportion of Receiving Any Therapy Services from Beta Regression Models – Rehabilitation Algorithm 4 or 5

|  | March 2020 | | | April 2020 | | |
| --- | --- | --- | --- | --- | --- | --- |
|  | Estimate | Standard error | p-value | Estimate | Standard error | p-value |
| β_2_ (pandemic-associated change in level) | -0.411 | 0.164 | 0.01 | -0.599 | 0.286 | 0.04 |
| β_3_ (pandemic-associated change in slope) | 0.031 | 0.011 | 0.006 | 0.041 | 0.017 | 0.02 |

Parameter Estimates for Adjusted Monthly Amount of Therapy from Linear Regression Models – Rehabilitation Algorithm 4 or 5

|  | March 2020 | | | April 2020 | | |
| --- | --- | --- | --- | --- | --- | --- |
|  | Estimate | Standard error | p-value | Estimate | Standard error | p-value |
| β_2_ (pandemic-associated change in level) | -0.825 | 0.233 | <0.001 | -0.578 | 0.408 | 0.16 |
| β_3_ (pandemic-associated change in slope) | 0.067 | 0.015 | <0.001 | 0.050 | 0.024 | 0.04 |
